# Age- and sex-based risk stratification in takotsubo syndrome: a Japanese nationwide registry study

**DOI:** 10.3389/fcvm.2026.1852892

**Published:** 2026-07-01

**Authors:** Tomohiro Hayashi, Kazuaki Negishi, Takato Kobayashi, Koshiro Kanaoka, Hozuka Akita, Yuto Shinkura, Ryo Nishio, Kensuke Matsumoto, Masahiko Hoshijima, Satoru Kawasaki, Hogara Nishisaki, Masanobu Okayama, Tsuneaki Kenzaka

**Affiliations:** 1 Division of Community Medicine and Career Development, Kobe University Graduate School of Medicine, Kobe, Japan; 2 Department of Internal Medicine, Hyogo Prefectural Tamba Medical Center, Tamba, Japan; 3 School of Clinical Medicine, University of New South Wales, Sydney, NSW, Australia; 4 Department of Cardiology, Liverpool Hospital, Liverpool, NSW, Australia; 5 The Ingham Institute for Applied Medical Research, Sydney, NSW, Australia; 6 Victor Chang Cardiac Research Institute, Darlinghurst, NSW, Australia; 7 Department of Medical and Health Information Management, National Cerebral and Cardiovascular Center, Osaka, Japan; 8 Division of Community Medicine and Medical Education, Kobe University Graduate School of Medicine, Kobe, Japan

**Keywords:** age, sex, outcome, risk stratification, takotsubo syndrome

## Abstract

**Background:**

Despite sex-related differences in the clinical presentation and outcomes of takotsubo syndrome (TTS), the prognostic significance of age remains debated, and evidence on male patients is limited. This study elucidated age- and sex-specific differences in clinical characteristics and outcomes of patients with TTS using a large nationwide Japanese registry.

**Methods:**

We conducted a retrospective cohort study using the nationwide Japanese Registry of All Cardiac and Vascular Diseases–Diagnosis Procedure Combination database (2012–2022). Patients with the first TTS episode, identified by the International Classification of Diseases, 10th Revision codes, who underwent coronary angiography and met the Mayo Clinic diagnostic criteria were included. Clinical characteristics, in-hospital management, and outcomes were compared across age groups (≤50, 51–74, ≥75 years) and sex. Independent in-hospital mortality predictors were assessed using multivariable logistic regression.

**Results:**

Among 13,585 patients, 2,558 (18.8%) were men, and 523 (3.8%), 5,354 (39.4%), and 7,708 (56.7%) were aged ≤50, 51–74, and ≥75 years, respectively. Men more frequently had comorbidities (chronic kidney disease, chronic obstructive pulmonary disease, malignancies) and required intensive care, including catecholamine administration, mechanical circulatory support, and ventilatory assistance, compared with women. Adverse events increased with age in both sexes. Across age groups, in-hospital mortality was higher in men (≤50 years: 3.0%; 51–74 years: 4.3%; ≥75 years: 8.2%) than in women (≤50 years: 1.5%; 51–74 years: 1.5%; ≥75 years: 4.6%). Multivariable analysis identified older age [odds ratio (OR) 1.32 per 5 years; 95% confidence interval (CI) 1.26–1.39; *P* < 0.001] and male sex (OR 1.59; 95% CI 1.30–1.95; *P* < 0.001) as independent in-hospital mortality predictors.

**Conclusion:**

Older age and male sex were independent predictors of in-hospital mortality in patients with TTS. These findings may help guide risk assessment and clinical monitoring in TTS according to age and sex, although further prospective validation is warranted.

## 1 Introduction

Takotsubo syndrome (TTS) is an acute but reversible cardiac dysfunction form typically triggered by emotional or physical stress, first described in Japan in 1990 (1, 2). TTS incidence in the coronavirus disease era has been significantly higher than that in prepandemic periods (3). Despite many recent TTS research advances, evidence-based treatments remain lacking owing to a poor understanding of its underlying pathophysiology (4–6). Notably, TTS predominantly affects older postmenopausal women (7), yet several previous smaller studies have suggested age- (8–10) and sex-related (11–13) differences in the clinical presentation and outcomes in patients with TTS. Nevertheless, the effects of age on TTS prognosis remain controversial (8–10), and data on male patients are particularly limited, as this subgroup has been relatively underrepresented in European and North American cohorts compared with those from Japan (11–18). Consequently, age- and sex-related differences in TTS clinical characteristics and prognosis remain incompletely understood.

Here, we aimed to provide a comprehensive assessment of age- and sex-specific clinical profiles, in-hospital management, and outcomes among patients with TTS using data from a large-scale, nationwide, multicenter Japanese registry.

## 2 Methods

### 2.1 Data source

We used the Japanese Registry of All Cardiac and Vascular Diseases and the Diagnosis Procedure Combination (JROAD-DPC) database, a nationwide institutional registry managed by the Japanese Circulation Society (JCS) (19, 20), which contains administrative claims data collected from more than 1,000 JCS-certified (associated) training hospitals in Japan, including patient information such as age, sex, diagnoses (principal diagnosis, comorbidities, and complications) based on the International Statistical Classification of Diseases, 10th Revision (ICD-10) codes, prescribed medications, therapeutic procedures, length of hospital stay, and in-hospital mortality.

The study protocol complied with the Declaration of Helsinki. It was approved by the Institutional Review Board of Hyogo Prefectural Tamba Medical Center (approval No. 1075) and the Japanese Circulation Society (approval No. 2022-14). The requirement for individual informed consent was waived by the institutional review board.

### 2.2 Study design and patients

**Figure 1:**
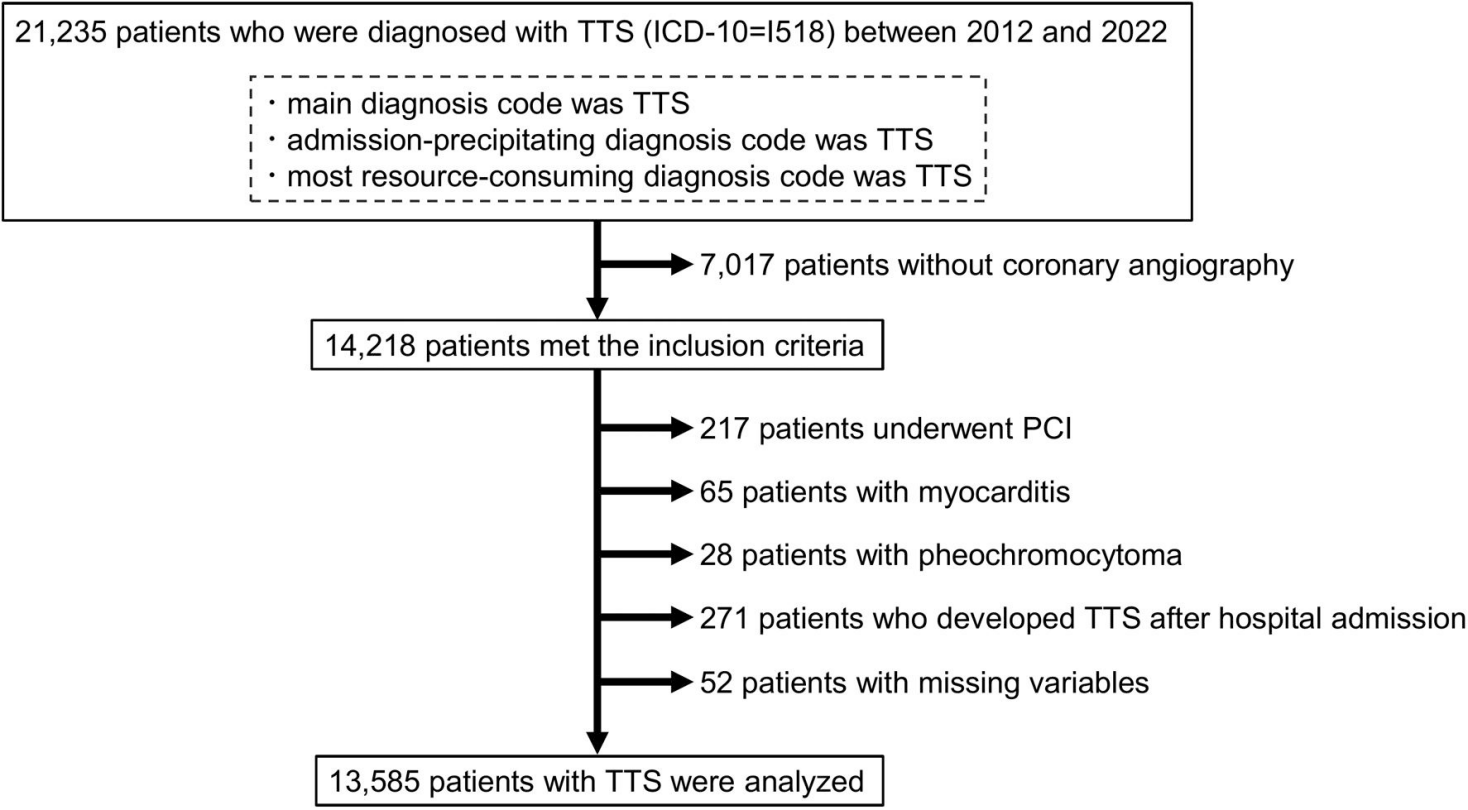
Study flowchart. Flow diagram illustrating patient selection from the JROAD-DPC database between April 2012 and March 2022. Patients with TTS who underwent coronary angiography were identified using ICD-10 codes in accordance with the Mayo Clinic diagnostic criteria. PCI, percutaneous coronary intervention; TTS, takotsubo syndrome; JROAD-DPC, Japanese registry of all cardiac and vascular diseases and the diagnosis procedure combination; ICD, international classification of diseases.

We conducted a retrospective cohort study using data from the JROAD-DPC database. Using the ICD-10 code, we extracted data regarding hospitalized patients between 1 April 2012 and 31 March 2022 with the first TTS episode who underwent coronary angiography and fulfilled the Mayo Clinic diagnostic criteria (21). The validity of the ICD-10 code for TTS (I518) in the JROAD-DPC database has been previously verified, demonstrating a sensitivity, specificity, and positive predictive value of 0.83, 1.00, and 1.00, respectively (22). Patients were excluded if they underwent percutaneous coronary intervention, were diagnosed with myocarditis (I40) or pheochromocytoma (D350), developed TTS following hospital admission, or had missing data for study variables. The final cohort comprised 13,585 patients with TTS from 1,141 hospitals across Japan (Figure 1).

### 2.3 Statistical analysis

Age was categorized into three clinically relevant groups (≤50, 51–74, and ≥75 years) to approximate premenopausal, postmenopausal, and older populations, based on previous literature (10). Categorical variables are described using numbers and percentages. Continuous variables are summarized using their mean and standard deviation. Patients with missing data for variables included in the analyses were excluded using complete-case analysis. Categorical variables were compared using the Pearson chi-square test. Continuous variables were analyzed using one-way analysis of variance and the Kruskal–Wallis test. Kaplan–Meier survival curves were constructed to analyze in-hospital mortality, and the log-rank test was used to compare groups. Univariable and multivariable logistic regression models were performed to determine odds ratios (ORs) and 95% confidence intervals (CIs) for variables associated with in-hospital mortality. Candidate variables for the multivariable model were prespecified based on clinical relevance and previous literature, and were further evaluated using univariable analyses before final model construction. Multicollinearity was assessed using variance inflation factors, and no evidence of significant collinearity among covariates was observed. Because the primary aim of the multivariable analysis was to identify independent associations with in-hospital mortality rather than to develop or validate a prediction model, formal assessments of model calibration and discrimination were not performed. All analyses were performed using JMP version 14.2 software (SAS Institute, Cary, NC, USA). A two-tailed *P* value <.05 was considered statistically significant.

## 3 Results

### 3.1 Clinical characteristics

Of 13,585 TTS patients included, 2,558 (18.8%) and 11,027 (81.2%) were men and women, respectively. Among male patients, 134 (5.2%), 998 (39.0%), and 1,426 (55.7%) were ≤50, 51–74, and ≥75 years of age, respectively. Among female patients, 389 (3.5%), 4,356 (39.5%), and 6,282 (57.0%) were ≤50, 51–74, and ≥75 years of age, respectively. Comparisons between groups according to age and sex are reported in Table 1.

**Table 1:** Characteristics of patients with TTS according to age and sex.

	Male TTS (*n* = 2558)			Female TTS (*n* = 11,027)			*P* values
	≤50 Years	51–74 Years	≥75 Years	≤50 Years	51–74 Years	≥75 Years	
	*n* = 134 (5.2%)	*n* = 998 (39.0%)	*n* = 1426 (55.7%)	*n* = 389 (3.5%)	*n* = 4356 (39.5%)	*n* = 6282 (57.0%)	
Age, years	42 ± 7	66 ± 6	82 ± 5	45 ± 6	66 ± 6	82 ± 5	<0.001
Comorbidities and cardiovascular risk factors							
Hypertension	65 (48.5%)	432 (43.3%)	608 (42.6%)	149 (38.3%)	1911 (43.9%)	3015 (48.0%)	<0.001
Diabetes mellitus	12 (9.0%)	192 (19.2%)	305 (21.4%)	35 (9.0%)	609 (14.0%)	966 (15.4%)	<0.001
Dyslipidemia	42 (31.3%)	216 (21.6%)	280 (19.6%)	73 (18.8%)	1355 (31.1%)	1714 (27.3%)	<0.001
COPD	0 (0%)	49 (4.9%)	96 (6.7%)	1 (0.3%)	28 (0.6%)	57 (0.9%)	<0.001
CKD	5 (3.7%)	76 (7.6%)	126 (8.8%)	7 (1.8%)	159 (3.7%)	284 (4.5%)	<0.001
Hypothyroidism	2 (1.5%)	8 (0.8%)	15 (1.1%)	5 (1.3%)	82 (1.9%)	107 (1.7%)	0.089
Hyperthyroidism	0 (0%)	1 (0.1%)	6 (0.4%)	11 (2.8%)	53 (1.2%)	50 (0.8%)	<0.001
Malignancies	2 (1.5%)	116 (11.6%)	198 (13.9%)	9 (2.3%)	220 (5.1%)	353 (5.6%)	<0.001
Neurological disorders							
Seizure	4 (3.0%)	26 (2.6%)	27 (1.9%)	12 (3.1%)	92 (2.1%)	76 (1.2%)	<0.001
TIA	0 (0%)	3 (0.3%)	3 (0.2%)	0 (0%)	6 (0.1%)	6 (0.1%)	0.530
Cerebral infarction	2 (1.5%)	44 (4.4%)	40 (2.8%)	3 (0.8%)	81 (1.9%)	188 (3.0%)	<0.001
Intracranial hemorrhage	0 (0%)	3 (0.3%)	8 (0.6%)	2 (0.5%)	14 (0.3%)	27 (0.4%)	0.751
Primary headache disorders^a^	1 (0.7%)	1 (0.1%)	0 (0%)	5 (1.3%)	9 (0.2%)	2 (0%)	<0.001
Psychiatric disorders							
Affective disorder	6 (4.5%)	20 (2.0%)	14 (1.0%)	9 (2.3%)	105 (2.4%)	145 (2.3%)	0.013
Anxiety disorder	4 (3.0%)	9 (0.9%)	11 (0.8%)	10 (2.6%)	91 (2.1%)	117 (1.9%)	0.003
Schizophrenia	1 0.7(%)	15 (1.5%)	22 (1.5%)	9 (2.3%)	67 (1.5%)	78 (1.2%)	0.449
Medications during hospitalization							
Antiplatelet drugs	66 (49.3%)	583 (58.4%)	888 (62.3%)	140 (36.0%)	2225 (51.1%)	3505 (55.8%)	<0.001
ACE inhibitors/ARBs	50 (37.3%)	390 (39.1%)	587 (41.2%)	103 (26.5%)	1465 (33.6%)	2508 (39.9%)	<0.001
Beta-blockers	25 (18.7%)	193 (19.3%)	317 (22.2%)	52 (13.4%)	774 (17.8%)	1366 (21.7%)	<0.001
Calcium channel blockers	47 (35.1%)	349 (35.0%)	491 (34.4%)	111 (28.5%)	1063 (24.4%)	1859 (29.6%)	<0.001
Loop diuretics	16 (11.9%)	260 (26.1%)	521 (36.5%)	58 (14.9%)	815 (18.7%)	2189 (34.8%)	<0.001
Spironolactone	8 (6.0%)	107 (10.7%)	214 (15.0%)	17 (4.4%)	386 (8.9%)	994 (15.8%)	<0.001
Statins	28 (20.9%)	252 (25.3%)	344 (24.1%)	55 (14.1%)	1242 (28.5%)	1865 (29.7%)	<0.001

Values are presented as numbers (%). ^a^Including migraine, tension-type, and cluster headaches. ACE, angiotensin-converting enzyme; ARB, angiotensin II receptor blocker; CKD, chronic kidney disease; COPD, chronic obstructive pulmonary disease; TIA, transient ischemic attack; TTS, takotsubo syndrome.

The prevalence of diabetes mellitus (male: 9.0%, 19.2%, and 21.4% vs. female: 9.0%, 14.0%, and 15.4%), chronic obstructive pulmonary disease (COPD; male: 0%, 4.9%, and 6.7% vs. female: 0.3%, 0.6%, and 0.9%), and chronic kidney disease (CKD; male: 3.7%, 7.6%, and 8.8% vs. female: 1.8%, 3.7%, and 4.5%) was higher in male patients with TTS than in female patients with TTS. Middle-aged and older male patients with TTS more frequently had malignancies compared with female patients with TTS of the same age groups (male: 11.6% and 13.9% vs. female: 5.1% and 5.6%, respectively).

Younger patients with TTS had seizures more frequently than did middle-aged and older patients (male: 3.0% vs. 2.6% vs. 1.9%; female: 3.1% vs. 2.1% vs. 1.2%). There were no significant differences in the prevalence of transient ischemic attack and intracranial hemorrhage among groups.

Among male patients with TTS, the younger group more often displayed psychiatric disorders, such as affective and anxiety disorders, than did the older groups (affective disorder: 4.5% vs. 2.0% vs. 1.0%, anxiety disorder: 3.0% vs. 0.9% vs. 0.8%). No significant difference in the prevalence of schizophrenia was observed among the groups.

In both male and female patients with TTS, younger individuals were less likely to receive medications during hospitalization, including angiotensin-converting enzyme inhibitors/angiotensin II receptor blockers (male: 37.3% vs. 39.1% vs. 41.2%, female: 26.5% vs. 33.6% vs. 39.9%), beta-blockers (male: 18.7% vs. 19.3% vs. 22.2%, female: 13.4% vs. 17.8% vs. 21.7%), loop diuretics (male: 11.9% vs. 26.1% vs. 36.5%, female: 14.9% vs. 18.7% vs. 34.8%), spironolactone (male: 6.0% vs. 10.7% vs. 15.0%, female: 4.4% vs. 8.9% vs. 15.8%), and statins (male: 20.9% vs. 25.3% vs. 24.1%, female: 14.1% vs. 28.5% vs. 29.7%), compared with older individuals.

### 3.2 Outcomes

During hospitalization, male patients with TTS more frequently required acute cardiac care treatments, including catecholamine use (male: 23.1% vs. 28.4% vs. 29.1%, female: 18.8% vs. 17.9% vs. 23.9%), mechanical circulatory support such as intra-aortic balloon pump and extracorporeal membrane oxygenation (male: 6.0% vs. 3.2% vs. 2.7%, female: 2.6% vs. 1.8% vs. 1.7%), and/or non-invasive and invasive ventilation (male: 12.7% vs. 17.2% vs. 16.3%, female: 9.8% vs. 6.6% vs. 10.4%), than did their female counterparts, as displayed in Table 2. The prevalence of ventricular arrhythmia (male: 3.0% vs. 2.2% vs. 1.7%, female: 3.6% vs. 0.8% vs. 1.1%), cardiogenic shock (male: 3.0% vs. 2.5% vs. 2.6%, female: 1.5% vs. 1.9% vs. 1.9%), and/or cardiopulmonary resuscitation (male: 3.7% vs. 1.1% vs. 0.5%, female: 2.1% vs. 0.2% vs. 0.2%) was also higher in male than in female patients with TTS. Male patients with TTS more often developed acute heart failure (male: 2.2% vs. 3.3% vs. 4.3%, female: 1.5% vs. 2.5% vs. 4.0%), had higher in-hospital mortality rates (male: 3.0% vs. 4.3% vs. 8.2%, female: 1.5% vs. 1.5% vs. 4.6%), and/or had longer hospital stays (male: 12 ± 17 vs. 14 ± 15 vs. 18 ± 17, female: 11 ± 13 vs. 12 ± 13 vs. 16 ± 15) than did their female counterparts, and these adverse events were more common in older patients than in younger patients.

**Table 2:** In-hospital complications, management, and outcomes in patients with TTS according to age and sex .

	Male TTS (*n* = 2558)			Female TTS (*n* = 11,027)			*P* values
	≤50 Years	51–74 Years	≥75 Years	≤50 Years	51–74 Years	≥75 Years	
	*n* = 134 (5.2%)	*n* = 998 (39.0%)	*n* = 1426 (55.7%)	*n* = 389 (3.5%)	*n* = 4356 (39.5%)	*n* = 6282 (57.0%)	
Infection	2 (1.5%)	63 (6.3%)	141 (9.9%)	13 (3.3%)	149 (3.4%)	382 (6.1%)	<0.001
Acute HF	3 (2.2%)	33 (3.3%)	62 (4.3%)	6 (1.5%)	109 (2.5%)	254 (4.0%)	<0.001
NE	23 (17.2%)	160 (16.0%)	225 (15.8%)	41 (10.5%)	479 (11.0%)	857 (13.6%)	<0.001
DOA	2 (1.5%)	65 (6.5%)	102 (7.2%)	16 (4.1%)	167 (3.8%)	357 (5.7%)	<0.001
DOB	6 (4.5%)	58 (5.8%)	88 (6.2%)	16 (4.1%)	132 (3.0%)	285 (4.5%)	<0.001
IABP	5 (3.7%)	27 (2.7%)	32 (2.2%)	7 (1.8%)	68 (1.6%)	92 (1.5%)	0.013
ECMO	3 (2.2%)	5 (0.5%)	7 (0.5%)	3 (0.8%)	9 (0.2%)	15 (0.2%)	<0.001
Impella	1 (0.7%)	3 (0.3%)	0 (0%)	0 (0%)	1 (0%)	1 (0%)	<0.001
Invasive ventilation	17 (12.7%)	165 (16.5%)	212 (14.9%)	37 (9.5%)	277 (6.4%)	597 (9.5%)	<0.001
Non-invasive ventilation	0 (0%)	7 (0.7%)	20 (1.4%)	1 (0.3%)	10 (0.2%)	56 (0.9%)	<0.001
VT/VF	4 (3.0%)	22 (2.2%)	24 (1.7%)	14 (3.6%)	33 (0.8%)	71 (1.1%)	<0.001
Cardiogenic shock	4 (3.0%)	25 (2.5%)	37 (2.6%)	6 (1.5%)	83 (1.9%)	118 (1.9%)	0.348
Cardiopulmonary resuscitation	5 (3.7%)	11 (1.1%)	7 (0.5%)	8 (2.1%)	10 (0.2%)	10 (0.2%)	<0.001
Stroke	2 (1.5%)	11 (1.1%)	12 (0.8%)	3 (0.8%)	26 (0.6%)	90 (1.4%)	0.002
In-hospital mortality	4 (3.0%)	43 (4.3%)	117 (8.2%)	6 (1.5%)	64 (1.5%)	292 (4.6%)	<0.001
Death within 7 days	3 (2.3%)	16 (1.6%)	45 (3.2%)	5 (1.3%)	39 (0.9%)	166 (2.6%)	<0.001
Death within 30 days	4 (3.0%)	37 (3.7%)	92 (6.5%)	6 (1.5%)	57 (1.3%)	249 (4.0%)	<0.001
Length of hospital stay (days)	12 ± 17	14 ± 15	18 ± 17	11 ± 13	12 ± 13	16 ± 15	<0.001

Values are numbers (%). DOA, dopamine; DOB, dobutamine; ECMO, extracorporeal membrane oxygenation; HF, heart failure; IABP, intra-aortic balloon pump; NE, norepinephrine; TTS, takotsubo syndrome; VF, ventricular fibrillation; VT, ventricular tachycardia.

**Figure 2:**
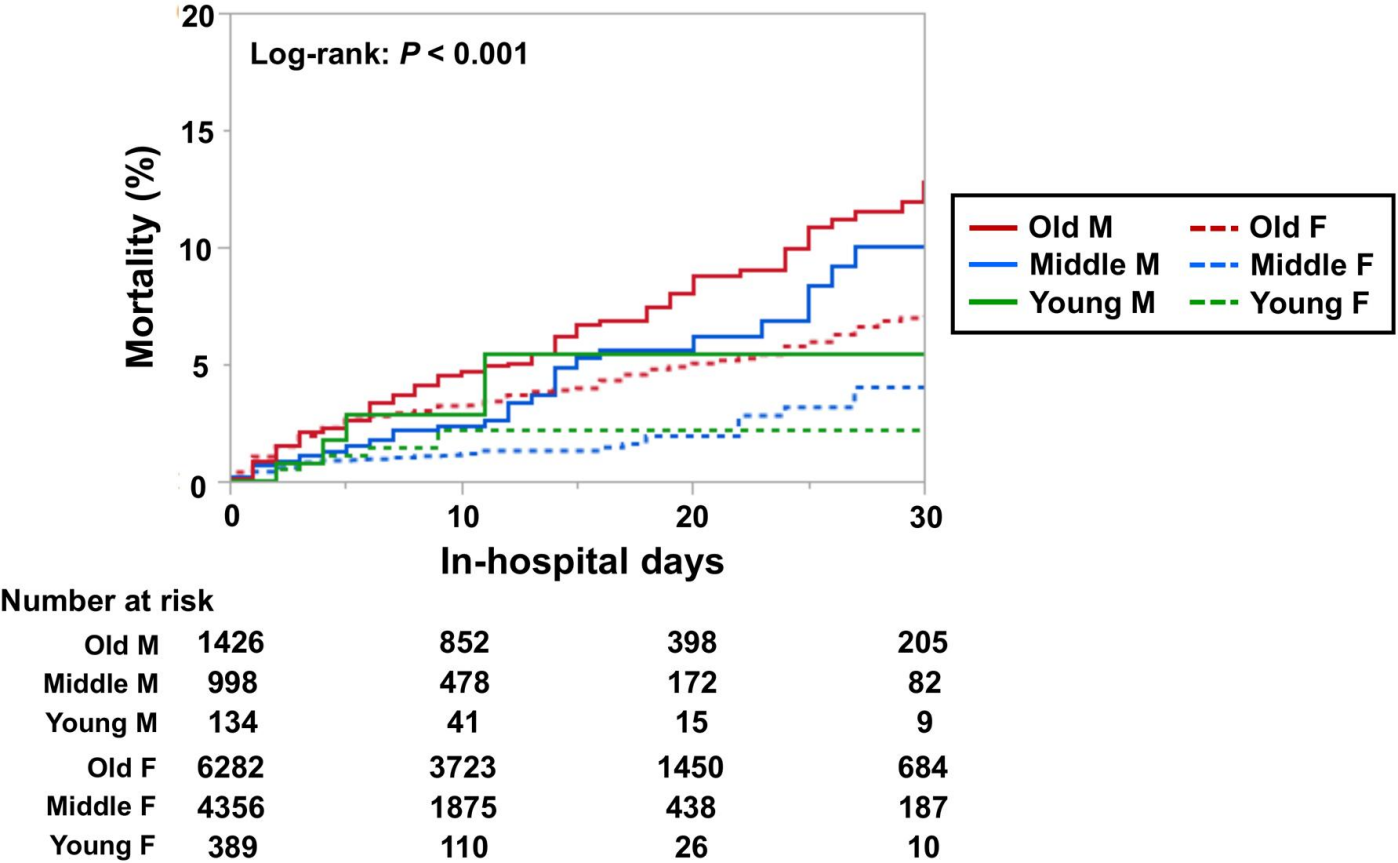
Kaplan-Meier curves for 30-day in-hospital mortality stratified by age and sex. Kaplan–Meier estimates of 30-day in-hospital mortality are presented for six groups defined by age (Young: ≤50 years, Middle: 51–74 years, Old: ≥75 years) and sex. F, female; M, male.

Kaplan–Meier survival curves of the six groups, stratified by age and sex, for 30-day in-hospital mortality are shown in Figure 2. Male patients with TTS had a higher in-hospital mortality than did their female counterparts of the same age groups. Moreover, older groups exhibited a higher in-hospital mortality compared with younger and middle-aged groups in both sexes (*P* < 0.001).

In the multivariable logistic regression analysis (Table 3), older age (OR: 1.32 per 5-year increase; 95% CI: 1.26–1.39; *P* < 0.001) and male sex (OR: 1.59; 95% CI: 1.30–1.95; *P* < 0.001) were independently associated with in-hospital mortality. Moreover, cardiogenic shock (OR: 4.37; 95% CI: 3.07–6.24; *P* < 0.001), malignancies (OR: 2.92; 95% CI: 2.30–3.72; *P* < 0.001), infection (OR: 3.26; 95% CI: 2.56–4.17; *P* < 0.001), neurological disorders (OR: 1.68; 95% CI: 1.20–2.36; *P* = 0.005), hypertension (OR: 0.39; 95% CI: 0.32–0.48; *P* < 0.001), and psychiatric disorders (OR: 0.40; 95% CI: 0.22–0.75; *P* = 0.001) remained independent predictors of in-hospital mortality.

**Table 3:** Univariable and multivariable logistic regression analyses for in-hospital mortality.

	Univariable		Multivariable	
	OR (95% CI)	*P* values	OR (95% CI)	*P* values
Age (per 5-year increase)	1.31 (1.26–1.39)	<0.001	1.32 (1.26–1.39)	<0.001
Male	2.02 (1.67–2.44)	<0.001	1.59 (1.30–1.95)	<0.001
Hypertension	0.37 (0.30–0.45)	<0.001	0.39 (0.32–0.48)	<0.001
Diabetes mellitus	1.01 (0.80–1.29)	0.907	−	−
COPD	2.83 (1.82–4.39)	<0.001	NS	NS
Malignancies	3.71 (2.95–4.65)	<0.001	2.92 (2.30–3.72)	<0.001
Neurological disorders	1.77 (1.28–2.45)	<0.001	1.68 (1.20–2.36)	0.005
Psychiatric disorders	0.38 (0.21–0.70)	0.002	0.40 (0.22–0.75)	0.001
Infection	4.48 (3.55–5.65)	<0.001	3.26 (2.56–4.17)	<0.001
Cardiogenic shock	5.11 (3.66–7.15)	<0.001	4.37 (3.07–6.24)	<0.001

CI, confidence interval; COPD, chronic obstructive pulmonary disease; NS, not significant; OR, odds ratio.

## 4 Discussion

This large multicenter study of 13,585 hospitalized patients with the first TTS episode represents the most comprehensive analysis of age- and sex-related clinical characteristics and outcomes of TTS to date and yields three main findings. First, men and patients aged ≤50 years accounted for 18.8% and 3.8% of the overall TTS population, respectively. Second, compared with women, men with TTS more frequently required intensive acute care interventions and had higher in-hospital mortality. Third, in-hospital mortality increased progressively with age in both sexes, with older patients experiencing the highest mortality rates.

Although TTS is more common in older females, particularly after menopause, there is growing recognition that male patients with TTS tend to experience worse outcomes, including life-threatening arrhythmias such as ventricular tachycardia and ventricular fibrillation, the need for inotropic support, higher cardiogenic shock rates, and increased in-hospital mortality compared with their female counterparts (13, 16, 23–27). Consistent with the findings of previous studies, the present research demonstrated that male patients with TTS—who were more likely to have a history of COPD, CKD, and malignancies—exhibited higher rates of catecholamine use, invasive mechanical ventilation, in-hospital mortality, and prolonged hospital stay compared with women. The reasons underlying these sex-related disparities remain unclear, and the exact role of sex hormones in TTS pathophysiology in both female and male patients has not been fully clarified (14). In preclinical studies using TTS animal models, estrogen supplementation to ovariectomized female rats attenuated stress-induced cardiac dysfunction (28, 29). Furthermore, male mice with stress-induced reversible cardiomyopathy exhibited more severe and persistent left ventricular dysfunction accompanied by enhanced myocardial inflammation compared with female mice (30). Therefore, the cardioprotective effects of estrogen through its favorable influence on microvascular function (31, 32), anti-inflammatory properties (33–35), and sympatholytic effects (36, 37) may contribute to the improved outcomes observed in female patients with TTS (38).

Although age-related differences in the clinical outcomes of patients with TTS have been reported in smaller studies, the findings remain inconsistent (8–10, 39, 40). In accordance with our results, previous studies reported that older patients with TTS demonstrated a higher prevalence of in-hospital adverse events (8) and greater in-hospital mortality (8, 40) than did younger individuals. Conversely, Cammann et al. recently reported that younger patients more frequently experienced in-hospital complications, including invasive or non-invasive ventilation, catecholamine use, cardiogenic shock, and cardiopulmonary resuscitation (10). Nevertheless, in that study, younger patients had a significantly higher proportion of men than older patients, suggesting that sex-related factors rather than age *per se* may have predominantly contributed to outcome differences across age groups. Indeed, several studies have demonstrated that male patients with TTS tend to be younger at the index event than female patients (40, 41). Physiologically, advancing age in healthy adults is associated with increased resting systemic sympathetic nervous system activity and sympathetic tone in the heart (42, 43). Furthermore, aging is accompanied by progressive microvascular and endothelial function impairment, driven by oxidative stress, mitochondrial dysfunction, and chronic low-grade inflammation (44, 45). Collectively, these mechanisms may predispose older individuals—characterized by elevated resting sympathetic tone and microvascular dysfunction—to TTS development.

Given the absence of detailed clinical variables in the JROAD-DPC database, including left ventricular ejection fraction, biomarkers, trigger type (emotional vs. physical), presence of left ventricular outflow tract obstruction, and TTS anatomical subtype, our findings should be interpreted with caution because of potential residual confounding. We observed that male and older patients with TTS more frequently developed acute heart failure and required intensive care support. Although TTS is generally considered a transient and reversible cardiomyopathy with favorable recovery, patients presenting with acute heart failure or cardiogenic shock represent a clinically high-risk phenotype characterized by substantial hemodynamic compromise, increased management complexity, and higher mortality risk. Recent studies have demonstrated that TTS complicated by acute heart failure or cardiogenic shock is associated with adverse short- and long-term outcomes (46, 47). Therefore, the greater use of intensive care observed in male and older patients in the present cohort likely reflects a higher burden of severe ventricular dysfunction and hemodynamic instability, which may partly account for their worse in-hospital outcomes.

In addition to intrinsic cardiovascular vulnerability, differences in trigger profiles and extracardiac comorbidities may contribute to the poorer prognosis observed in male and older patients with TTS. Physical stressors are more common in male patients and are associated with worse short- and long-term mortality than emotionally triggered TTS (13, 48). Furthermore, secondary TTS is more prevalent among male and older patients and is associated with a higher incidence of cardiogenic shock and increased long-term mortality compared with primary TTS (49). Because the present database does not permit characterization of trigger type or differentiation between primary and secondary TTS, these factors should be considered important potential confounders in the interpretation of our findings. In the current cohort, male and older patients exhibited substantially higher rates of CKD, COPD, malignancies, infection, and intensive care utilization, suggesting that male sex and advanced age may, at least in part, serve as surrogate markers of greater systemic illness burden rather than solely reflecting intrinsic biological susceptibility. Accordingly, the observed sex- and age-related differences in TTS outcomes are likely multifactorial, reflecting complex interactions among comorbidity burden, trigger profile, and hemodynamic severity, rather than being attributable exclusively to hormonal influences or inherent myocardial vulnerability.

Intriguingly, the present study demonstrated that psychiatric disorders were associated with lower in-hospital mortality in patients with TTS. Previous studies have similarly reported that pre-existing psychiatric disorders were not associated with increased short- or long-term mortality in TTS (50, 51). The mechanisms underlying this association remain unclear; however, patients with psychiatric disorders may more commonly present with emotionally triggered TTS, a phenotype generally associated with more favorable outcomes than physically triggered TTS (48). Further large-scale studies including detailed psychiatric phenotyping and psychotropic medication data are warranted to clarify the associations among psychiatric disorders, treatment profiles, and TTS outcomes.

**Figure 3:**
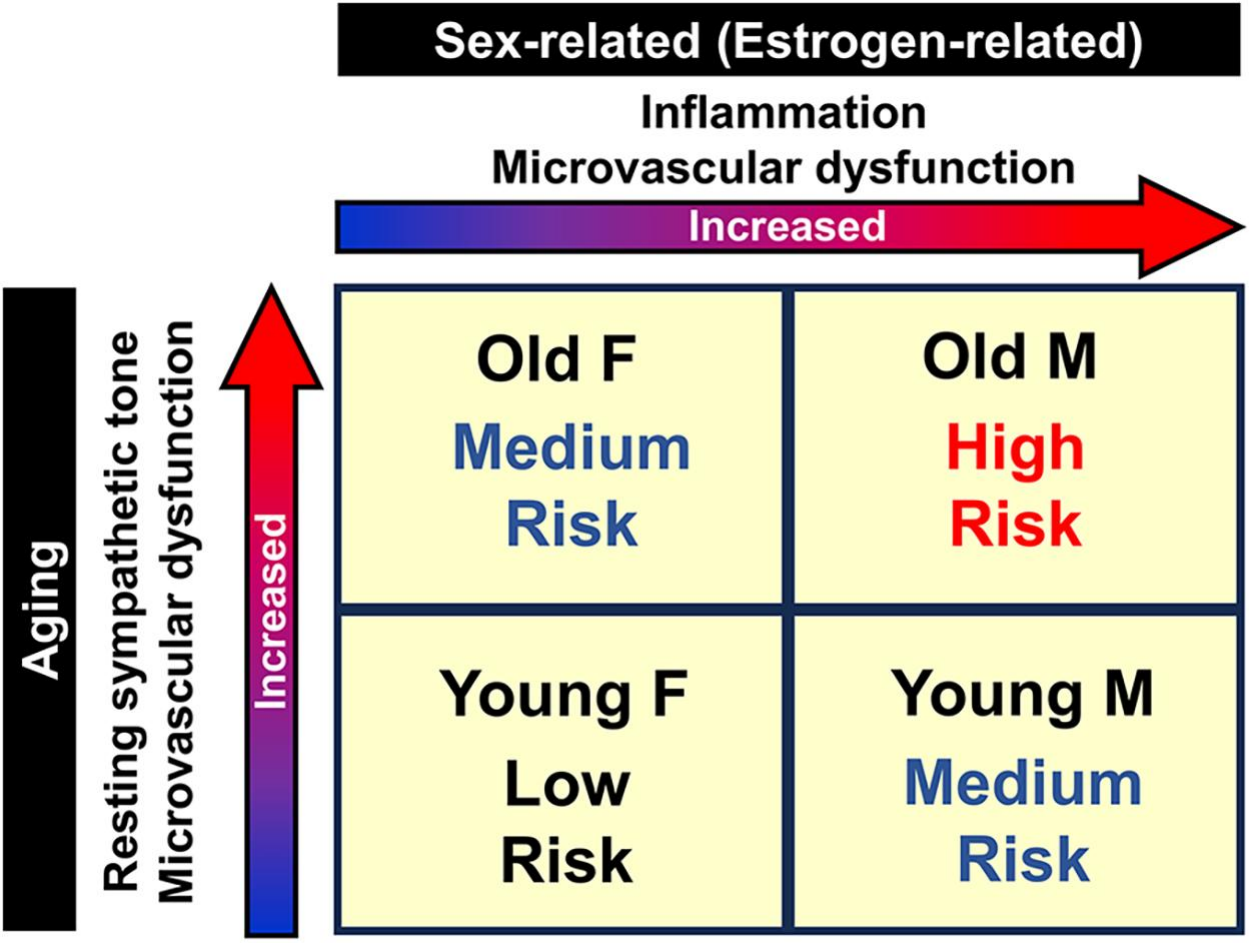
Conceptual framework for age- and sex-related differences in TTS outcomes. Older age and male sex were associated with worse in-hospital outcomes in the present study. Potential contributors to these differences may include increased sympathetic activity, microvascular dysfunction, inflammation, a greater extracardiac comorbidity burden, differences in trigger profile, and a higher prevalence of secondary TTS. Because detailed clinical variables and trigger characteristics were unavailable in the present database, this framework should be considered hypothesis-generating rather than a validated clinical risk stratification model. F, female; M, male; TTS, takotsubo syndrome.

Based on the present findings and prior experimental and clinical evidence, we propose a conceptual framework for understanding age- and sex-related differences in TTS outcomes (Figure 3). Because the present study was based on an observational administrative database lacking detailed mechanistic and phenotypic information, this framework should be regarded as hypothesis-generating rather than a validated clinical risk stratification tool. Further prospective studies incorporating detailed clinical, imaging, and biomarker data are warranted to validate these associations and clarify the underlying mechanisms.

This study has several limitations. First, this was a retrospective observational study and therefore subject to inherent biases. Second, the JROAD-DPC is an anonymized administrative database that lacks detailed clinical data, including vital signs, laboratory measurements, and echocardiographic findings. Consequently, we were unable to assess triggering factors, differentiate between primary and secondary forms of TTS, or characterize TTS subtypes based on the distribution of left ventricular regional wall motion abnormalities. In addition, because the database did not include information regarding the causes of in-hospital death, we could not distinguish cardiovascular from non-cardiovascular mortality. Finally, because post-discharge information was unavailable, long-term outcomes could not be evaluated.

In conclusion, among 13,585 patients with TTS, 523 (3.8%), 5,354 (39.4%), and 7,708 (56.7%) were aged ≤50, 51–74, and ≥75 years, respectively, and 2,558 (18.8%) were men. Male sex and advanced age were independently associated with worse in-hospital outcomes among patients with TTS in this large nationwide cohort. These findings highlight substantial heterogeneity in clinical presentation and prognosis according to age and sex and may help inform risk assessment and clinical monitoring in patients with TTS. However, given the observational design and limited granularity of the database, the proposed age- and sex-related framework should be interpreted as hypothesis-generating and requires further validation in prospective studies with detailed clinical and mechanistic characterization.

## Data Availability

The data analyzed in this study is subject to the following licenses/restrictions: The data underlying this study were obtained from the Japanese Circulation Society under a data-use agreement and cannot be made publicly available. Access to the data may be granted upon reasonable request to the corresponding author and with approval from the Japanese Circulation Society. Requests to access these datasets should be directed to Tomohiro Hayashi, tomohiro884@hotmail.com.
